# ZiBu PiYin recipe prevents diabetes-associated cognitive decline in rats: possible involvement of ameliorating mitochondrial dysfunction, insulin resistance pathway and histopathological changes

**DOI:** 10.1186/s12906-016-1177-y

**Published:** 2016-07-08

**Authors:** Zheng Sun, Libin Zhan, Lina Liang, Hua Sui, Luping Zheng, Xiaoxin Sun, Wei Xie

**Affiliations:** Institute of Integrative Medicine, Dalian Medical University, No. 9, South Road of Lvshun, Dalian, China; Nanjing University of Chinese Medicine, 138 Linxian Road, Nanjing, 210023 Jiangsu P.R. China; The Second Affiliated Hospital of Dalian Medical University, Dalian, 116023 Liaoning P.R. China

**Keywords:** DACD, ZBPYR, Mitochondrial dysfunction, Insulin resistance, Histopathological changes

## Abstract

**Background:**

Disturbance in energy metabolism, as a key factor in diabetes-associated cognitive decline (DACD), has become a promising therapeutic target of Chinese medicine ZiBu PiYin Recipe (ZBPYR). However, it is still not clear how ZBPYR affects the mitochondrial function in DACD rats’ brains, which is considered as the crucial cell organelle to supply energy for the brain.

**Methods:**

Type 2 diabetes mellitus (T2DM) rat models were established by using high fat diet and streptozotocin (STZ) (30 mg/kg, ip). The evaluation of insulin sensitivity was performed by oral glucose tolerance and insulin tolerance test. After 7 weeks, the T2DM rats were treated with vehicle or ZBPYR for 11 weeks and morris water maze (MWM) test were used to evaluate memory function. The ultra structural changes of prefrontal cortex (PFC) and hippocampus were examined by transmission electron microscopy (TEM). The mitochondrial membrane potential (ΔΨm) and reactive oxygen species (ROS) were measured with JC-1 and DCFDA assay. The levels of insulin proteins were quantified by Western Blot analysis and the markers of histopathological changes were detected by immunohistochemistry.

**Results:**

ZBPYR could alleviate learning and memory impairment of DACD rats. TEM showed that ZBPYR prevented mitochondrial ultra-structural alterations and number changes in the PFC and hippocampus of the DACD rats. In addition, ZBPYR significantly increased ΔΨm and lowered the levels of ROS. Further investigation indicated that ZBPYR suppressed the release of cytochrome c from mitochondria, strengthened insulin signaling and inhibited GSK3β over-expression. These positive effects were associated with reduced Aβ_1-42_ deposition and restored expression levels of microtubule-associated protein MAP2.

**Conclusion:**

ZBPYR showed excellent protective effect against DACD via ameliorating mitochondrial dysfunction, insulin resistance and histopathological changes.

## Background

Diabetes-associated cognitive decline (DACD) is diabetic complication, due to changes in the central nervous system (CNS) [1]. Considerable attention has been given to DACD in the last decade [2]. Previous studies have shown that cerebrovascular changes, oxidative stress, increased expression of advanced glycation end products (AGEs) and impaired cerebral insulin signaling are the underlying causes of DACD [3–6]. Our recent data have demonstrated that defection in energy metabolism of hippocampus might affect the function of the brain. It might be a key determinant of DACD [7].

Mitochondria are considered as crucial cell organelle that produces energy for regulating the cellular metabolism. It has been reported that brain mitochondria regulate energy-demanding neuro-transmission and calcium homeostasis, which are important mechanisms for the learning and memory process [8, 9]. In recent years, it has become increasingly clear that there is correlation between brain mitochondrial dysfunction and several neurodegenerative diseases [10]. Mitochondrial dysfunction is an important contributor to the damage and loss of neurons [11]. It has been also found that there was significant decrease in neuron numbers in the cerebral cortex or hippocampus in diabetic cognitive decline rats [12, 13]. Therefore, preventing mitochondrial dysfunctions is taken as a new therapeutic target for DACD.

Traditional Chinese medicine (TCM) has a long history in China. The ZiBu PiYin recipe (ZBPYR), derived from a modification of the Zicheng Decoction, is a TCM recorded in the book of Bujuji by Cheng Wu in the Qing Dynasty [14]. Early reports demonstrated that ZBPYR protected glutamate induced neuron injury and improved learning and memory of pi-yin deficiency diabetic rats and type 2 diabetic rats [7, 14, 15]. Recent findings have proved that ZBPYR could protect db/db mice from reducing dendritic spine density, attenuated brain leptin and insulin signaling pathway injury [16]. The potential effects of ZBPYR on the mitochondrial function of the brain in DACD model have not been investigated yet. In the present work, we used high-fat diet combined with Streptozotocin (STZ) rats as DACD model, aiming to test the hypothesis that ZBPYR could reverse the impairment of DACD mainly though alleviating brain mitochondrial dysfunction.

## Methods

### Tested drug and preparation

The ingredients of ZBPYR are: Red Ginseng, Common Yam Rhizome, Indian Buead, White Peony Root, Dan shen Root, White Hyacinth Bean, Lotus Seed, Grassleaf Sweetflag Rhizome, Thinleaf Milkwort Root, Sandalwood, Tangerine Red Epicarp and Liquorice Root. All herbs were purchased from Dalian Metro Pharmaceutical Co., Ltd. (Dalian, Liaoning Province, China) and authenticated by Prof. Yunpeng Diao (College of Pharmacy, Dalian Medical University). Voucher Specimens were deposited at the authors’ laboratory (Table 1). The herb mixtures (164.5 g) were soaked in 8 volumes v/w of distilled water for 30 min and then boiled for 90 min [17]. The decoction was then filtered through six-layer gauzes and made to a concentration of 1 g crude drug/ml, and finally the decoction was transformed into the freeze-dried powder. A 91.2 g of the freeze-dried powder was extracted with 10 mL distilled water (9.12 g/mL) for 30 min under ultrasound and stored at 4 °C before use.

**Table 1:** The ingredients of Zibu Piyin Recipe

Chinese name	Botanical Latin name	Plant part used	Weight (g)	Voucher numbers
Hong-Shen	*Panax ginseng* C.A.Mey.	Red Ginseng Root	30	ZBPYR01-120920
Shan-Yao	*Dioscorea polystachya* Turcz.	Common Yam Rhizome	15	ZBPYR02-120920
Fu-Ling	*Wolfiporia extensa* (Peck) Ginns	Indian Buead	15	ZBPYR03-120920
Bai-Shao	*Paeonia lactiflora* Pall.	White Peony Root	15	ZBPYR04-120920
Dan-Shen	*Salvia miltiorrhiza* Bunge	Dan shen Root	12	ZBPYR05-120920
Bai-Bian-dou	*Lablab purpureus* (L.) Sweet	White Hyacinth Bean	15	ZBPYR06-120920
Lian-Zi	*Nelumbo nucifera* Gaertn.	Lotus Seed	20	ZBPYR07-120920
Shi-Chang-Pu	*Acorus gramineus* Sol. ex Aiton	Grassleaf Sweetflag Rhizome	10	ZBPYR08-120920
Yuan-Zhi	*Polygala tenuifolia* Willd.	Thinleaf Milkwort Root	10	ZBPYR09-120920
Tan-Xiang	*Santalum album* L.	Sandalwood	4.5	ZBPYR10-120920
Ju-Hong	*Citrus maxima* ‘Tomentosa’	Tangerine Red Epicarp	9	ZBPYR11-120920
Gan-Cao	*Glycyrrhiza uralensis* Fisch. ex DC.	Liquorice Root	9	ZBPYR12-120920

### Experimental animals

Male Sprague–Dawley (SD) rats (Six week-old, 200 ± 20 g) were purchased from the Experimental Animal Center of Dalian Medical University (Dalian, China). They were maintained at 22 ± 3 °C with 65 ± 5 % humidity on a 12 h light/dark cycle during the experiments. All surgery was performed under anesthesia with ether, and all efforts were made to minimize suffering.

### Experimental design

**Fig. 1:**
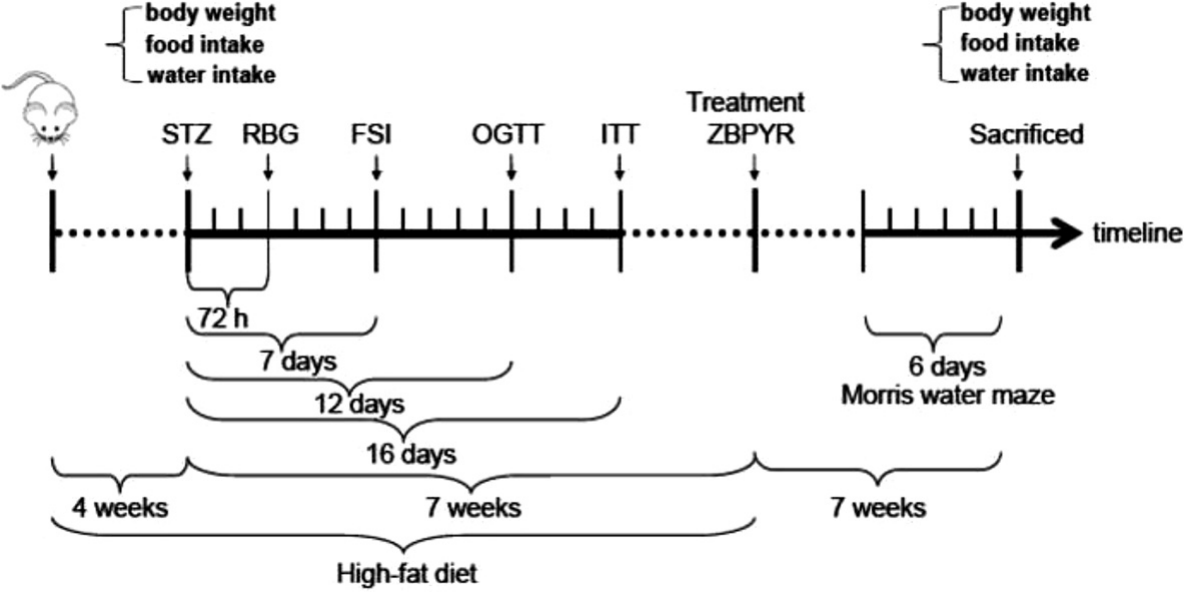
Design of the animal study. The control group was fed with standard diet during the whole study. The two diabetic groups received high-fat diet until drug intervention (ZBPYR). In week 4 of the timeline, the two diabetic groups received STZ, while the control group was administered vehicle citrate buffer. At 72 h, and 7, 12 and 16 days after STZ, RBG, FSI, OGTT and ITT were tested. From 7 weeks after the STZ injection, ZBPYR was administered to the ZBPYR group daily for 7 weeks. The Control and Model groups received physiological saline. The Morris water maze test was performed for 6 days before animals sacrificed

Thirty rats were randomly divided into three groups: citrate buffer injected standard diet rats (Control group); vehicle treated high-fat diet STZ rats (Model group); ZBPYR treated high-fat diet STZ rats (ZBPYR group). As shown in Fig. 1, high-fat diet (Anlimo Technology, Nanjing, China) were started 4 weeks prior to the STZ (Sigma, St Louis, MO, USA, 30 mg/kg, i.p) injection and continued for 11 weeks. The suspension of STZ was freshly dissolved in 0.1 M citrate buffer solution (Sigma, St Louis, MO, USA). Body weight, food intake and water intake of the rats were measured until 4 weeks high diet. Glucose levels in tail blood samples were determined using a glucometer (Accuchek; Roche, Mannheim, Germany) after STZ for 3 days. The fasting serum insulin (FSI), oral glucose tolerance test (OGTT) and insulin tolerance test (ITT) were performed at 7, 12 and 16 days after STZ injection. During a period of 7 weeks, ZBPYR group rats were orally administered ZBPYR at a dose of 0.1 ml/10 g body weight, while the Control and Model rats were orally administered an identical dose of ultrapure water (Milli-Q Integral Water Purification System, Millipore Corporation, Billerica, MA, USA). At the end of the experiment, body weight, food, water intake and red blood glucose (RBG) of the rats were measured among the three groups.

### Morris water maze test

Spatial learning and memory performance was assessed by morris water maze (MWM) test, which consisted of 5-day training (invisible plat training sessions and a probe trial) and visible plat training session on day 6. The apparatus consisted of a circular pool (120 cm in diameter, 50 cm in height and filled to a depth of 30 cm with water maintained at 28 ± 1 °C) and a transparent platform (9 cm in diameter and 29 cm in height), which was hidden under water. An automatic photographic was used to record the data (Institute of Materia Medica, Chinese Academy of Medical Sciences, Beijing, China). On the first day, rats were permitted to swim freely in the tank for 60 s without the platform to adapt to the new condition. Over the following 4 days, rats were trained with four trials per day at intervals of 120 s until they reached the platform and were allowed to rest for 20 s. If the rat failed to reach the platform within 120 s, it was gently guided to the platform and stayed for 20 s. The escape latency and swimming distance taken to reach the platform were measured automatically. After hidden platform test, the platform was removed. Rats had to swim for 120 s and the data referring % of total time in target quadrant. Time in searching original platforms and times across the platform were recorded. On the 6^th^ day, a visible-platform test was undertaken. The platform was located 2 cm over the water surface and placed at a position different from the previous test. During this test, the escape latency was recorded.

### Ultrastructural examination by transmission electron microscopy

4 % paraformaldehyde fixed prefrontal cortex (PFC) and hippocampus samples (3 mm × 3 mm) of Control, Model and ZBPYR groups were collected and fixed in 2 % glutaraldehyde in 0.1 mol/L sodium phosphate buffer (pH 7.4) overnight at 4 °C. After dehydration with a graded ethanol series, the sample was embedded in Epon812 and sectioned using a Leica EM UC6 ultramicrotome (Leica Co., Vienna, Austria). Samples were viewed and photographed using Transmission Electron Microscopy (TEM) (JEM-2000EX, JEDL, Japan).

### Preparation mitochondria of prefrontal cortex and hippocampus

The rats were sacrificed by an animal expert in accordance with approved ministerial procedures appropriate to the species. The PFC and hippocampus were quickly separated and the tissue blocks were finely minced in 8 μm thickness [18]. 50 pieces of sections were collected and homogenized in 1 mL isolation buffer according to the Tissue Mitochondria Isolation Kit (MitoSciences, Abcam, USA). The homogenate was centrifuged at 1000 g for 10 min at 4 °C and the supernatant was collected and further centrifuged at 12,000 g for 15 min at 4 °C. Then, mitochondrial pellets were collected, resuspending in 1 mL isolation buffer supplemented with 10 μL protease inhibitor cocktail (Sigma-Aldrich, St. Louis, MO, USA) and further centrifuged at 12,000 g for 15 min at 4 °C. Finally, the mitochondrial pellets were resuspended with 500 μL isolation buffer supplemented with protease inhibitor. The concentration of mitochondrial protein was measured by the BCA protein assay kit (cwbiotech, Beijing, China).

### Mitochondrial membrane potential assay

Changes in mitochondrial membrane potential (ΔΨm) were analyzed using a fluorescence spectrophotometer (PARADIGM, Molecular Devices, USA) and a mitochondria-selective dye (JC-1; Beyotime Institute of Biotechnology, Bejing, China). JC-1 was a lipophilic cation, driven by ΔΨm in normal polarized mitochondria assembled into a red fluorescence-emitting dimer forming J-aggregates. However, the monomeric form present in cells with depolarized mitochondrial membranes emited only green fluorescence.

Isolated mitochondria from PFC and hippocampus were stained with JC-1 staining solution (5 μg/mL) for 15 min at 37 °C and rinsed twice with assay buffer. The fluorescent intensity was analyzed on a spectrofluorometer as described to detect green fluorescence at excitation/emission wavelengths of 485/530 nm and red fluorescence at excitation/emission wavelengths of 550/595 nm. The ratio of red to green fluorescence intensity was determined for each sample as a measure of ΔΨm.

### Measurement of reactive oxygen species production

Reactive oxygen species (ROS) Assay Kit (Nanjing Jiancheng Bioengineering Institute, Nanjing, China) was used to quantify ROS levels of PFC and hippocampus tissues to detect the changes of mitochondrial mass. The fluorescent intensity of the tissue suspensions was measured by a fluorescence spectrophotometer (PARADIGM, Molecular Devices, USA) at excitation wavelength of 485 nm and emission wavelength of 520 nm.

### Protein extraction and western blotting analysis

The tissues of PFC and hippocampus were homogenized in ice cold lysis buffer [7]. Then, the homogenate was centrifuged at 15,000 g for 5 min at 4 °C prior to collect the supernatants. Protein concentrations in the supernatants were measured using the bicinchoninic acid (BCA) protein assay kit (cwbiotech, Beijing, China). 50 μg of the sample proteins was separated by electrophoresis in 10 % sodium dodecylsulfate-polycrylamide gel electrophoresis (SDS-PAGE) and transferred to polyvinylidenedi fluoride membrance (Amersham, Buckinghamshire, UK). The membrance was blocked with 5 % skimmed milk in TBS-T (10 mM Tris-Cl, PH 8.0, 150 mM NaCl and 0.5 % Tween 20) at 4 °C overnight. It was rinsed three times (10 min/time) with TBS-T, followed by 2 h incubation with the first antibodies in the appropriate concentrations and then 2 h incubation with HRP-conjugated secondary antibody. The immuno-labeling was detected using the enhanced chemiluminesence system (Roche, GmbH, Mannheim, Germany) and visualized using the UVP Bio-spectrum Imaging System (UVP, Inc, Upland, CA, USA). Primary antibodies used from Cell Signaling Technologies (Danvers, MA, USA) were: protein kinase B (Akt) (#9272, 1:800), p-Ser^473^Akt (#4058, 1:800), GSK3β (#9315, 1:800), p-Ser^9^GSK3β (#9322, 1:800) and microtubule-associated protein 2 (MAP2) (#4542, 1:800). Primary antibody directed against cytochrome c (Abcam, Cambridge, UK, #ab110325, 1:800), insulin receptor substrate 2 (IRS2) (Millipore Corporation, Billerica, MA, USA, #MABS15, 1:2000) and p-Ser^731^ IRS-2 (Abcam plc, Cambridge, UK, #ab3690, 1:200) were also used in the experiments. Goat anti-rabbit (#NA9340, 1:2000) or Goat anti-mouse (#NA9310, 1:2000) (GE Healthcare, Buckinghamshire, UK) were used as secondary antibodies. β-actin (Sigma-Aldrich, St Louis, MO, USA, #A2228, 1:2000) and Voltage-dependent anion channel (VDAC) (Cell Signaling Technologies, Danvers, MA, USA, #4866, 1:800) were used as total internal quantitative control and mitochondrial internal quantitative, respectively.

### Immunohistochemistry staining for Aβ_1-42_ and MAP2

Rats from each group were deeply anesthetized with pentobarbital sodium and perfused with 0.1 M PBS followed by 4 % paraformaldehyde. Brains were removed immediately and transferred to 15 %, 30 % sucrose solution (in PB) and then fixed in 4 % paraformaldehyde overnight at 4 °C. Sections of PFC and hippocampus were isolated and frozen on a cold stage, which was then cut at 6 μm according to the routine procedure [19]. Immunohistochemical staining was performed using rabbit anti-(Rat MAP2 IgG) at dilutions of 1:80 (Sigma-Aldrich, St Louis, MO, USA) and mouse anti-(Rat Aβ_1-42_ IgG) at dilutions of 1:80 (Abcamplc, Cambridge, UK), respectively. The sections without first antibody incubation were used as background control. The images of PFC and the CA1, CA3 and DG regions of the hippocampus were captured by an upright microscope (Leica, Wetzlar, Germany). The quantitative analysis was used by the “integrated optical density” (IOD) function of Image Pro-Plus version 6.0.

### Statistical analysis

Statistical analysis was performed using either an ANOVA (equal variance) or a Welch’s ANOVA (unequal variance) test. Data from the MWM test was analyzed using a repeated-measure analysis of variance for comparisons among trials. Student’s *t* test was used for comparison in two groups. The difference was considered to be statistically significant when *p* < 0.05.

## Results

### Effects of ZBPYR on type 2 diabetes models

**Fig. 2:**
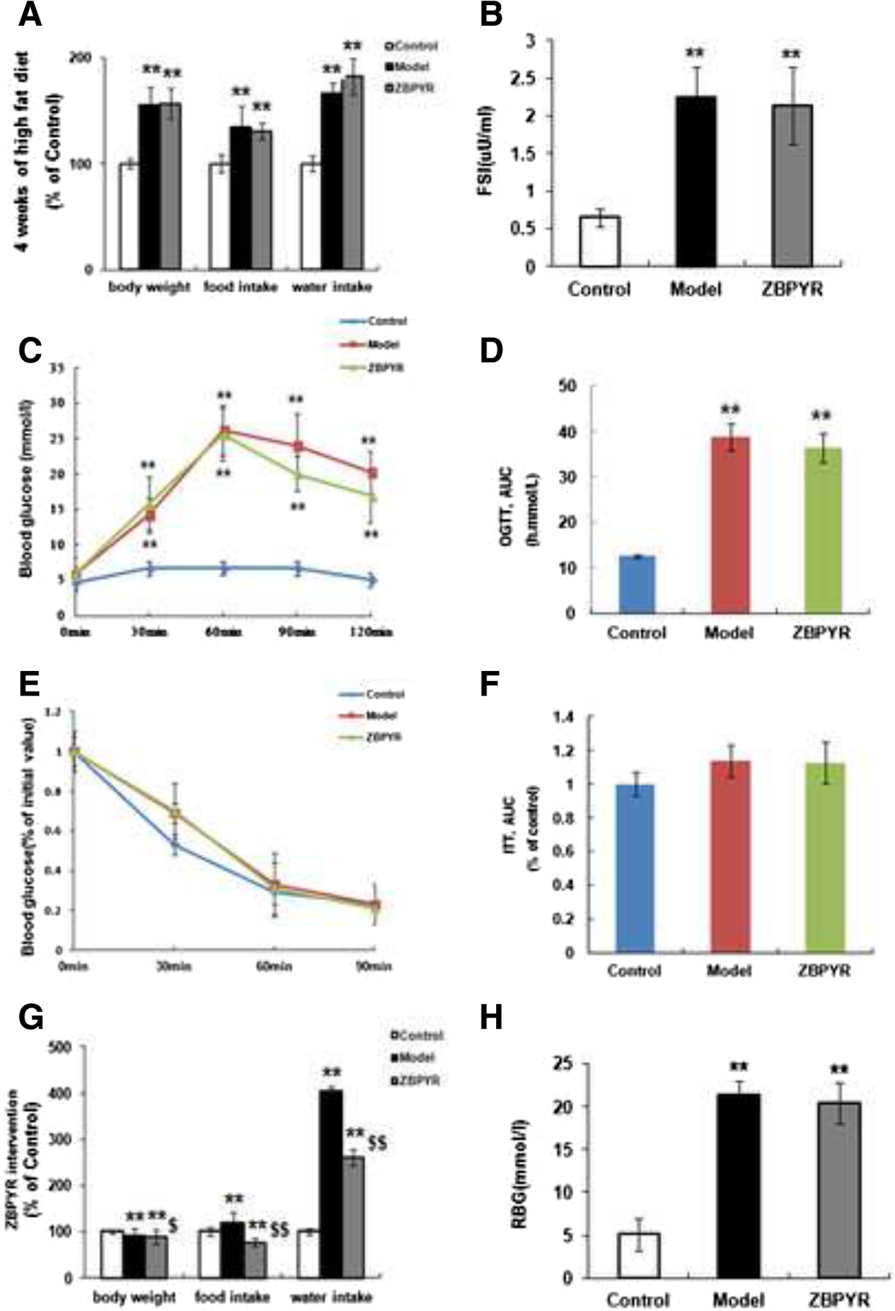
Diabetic rats was set up and treated by ZBPYR. **a** Body weight, food and water intake were measured after 4 weeks of high fat diet in Control, Model and ZBPYR rats. **b** FSI levels were determined 7 days after STZ injection. **c**-**d** OGTT was performed 12 days after STZ. Blood glucose levels were measured at the indicated times (**c**), and then the AUC was analyzed (**d**). **e**–**f** ITT was performed 16 days after STZ. Blood glucose levels were measured at the indicated time (**e**), and then the AUC was analyzed (**f**). **g** Body weight, food and water intake were measured after ZBPYR treatment in week 7. **h** RBG was detected after ZBPYR treatment in week 7. Mean ± S.E.M, *n* = 10. *Compared with Control group; ^$^ ZBPYR compared with Model group;*^, $^ *p* < 0.05, **^, $$^ *p* < 0.01

The results revealed that compared with the control group, the body weight, food and water intake of model rats were all increased rapidly due to the high-fat diet administered prior to the STZ injection (Fig. 2a). However, the FSI of model group were changed but not obviously (*p* > 0.05, data not shown). After STZ, FSI in model group was increased significantly (Fig. 2b). The levels of blood glucose of OGTT were significantly higher in model group than that of control group (Fig. 2c-d). ITT results were showed insulin sensitivity impaired in the model rats, but the group differences were not significantly (Fig. 2e-f). Following the injection of STZ, water and food intake were slowly increased. However, body weight in the model rats was decreased compared with the control group. ZBPYR treatment could make an obvious reduction in the food and water intake and body weight (Fig. 2g). But it had no obvious effects on blood glucose (Fig. 2h).

### Effects of ZBPYR on diabetes-induced cognitive decline

**Fig. 3:**
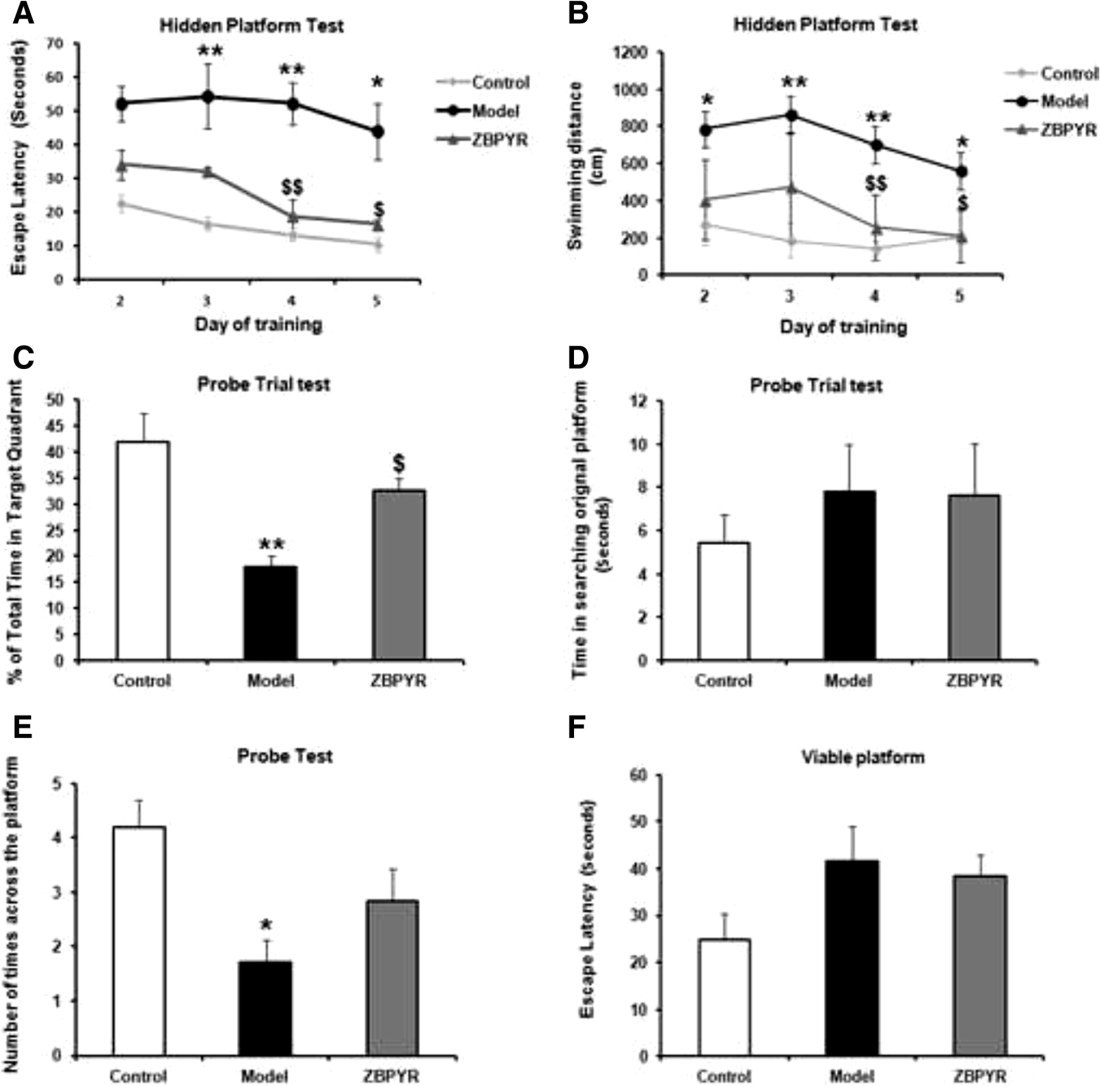
Effects of ZBPYR on DACD rats in morris water maze test. **a** Spatial memory acquisition phase, **b** swimming distance, **c** mean percentage of total time in the target quadrant, **d** time in searching original platforms, **e** the number of times crossing the platform and **f** escape latency to test visual acuity in Control, Model, and ZBPYR rats. Values are mean ± S.E.M, *n* = 10. *p < 0.05 and ** *p* < 0.01 vs. Control group; ^$^ *p* < 0.05 and ^$$^ *p* < 0.01 ZBPYR vs. Model group

In the hidden platform test, as shown in Fig. 3a, the escape latency of the model group was longer than that of the control group (days 2 to 4, *p* < 0.01; days 5, *p* < 0.05). In contrast, ZBPYR group required a short escape latency compared with model group (days 4, *p* < 0.01; days 5, *p* < 0.05). The swimming distance that the model rats reached the platform was significantly increased compared to the control group (days1, *p* < 0.05; days 2 to 4, *p* < 0.01; days 5, *p* < 0.05). After ZBPYR treatment, we found that the swimming distance was shorter than that of model rats (days 4, *p* < 0.01; days 5, *p* < 0.05) (Fig. 3b).

In the probe trial, the percentage of the total time that the model rats spent in the target quadrant was significantly lower than control group (Fig. 3c, *p* < 0.01). However, ZBPYR group performed differently compared with model group (Fig. 3c, *p* < 0.05). Time in searching original platform was also acquired by the probe trial test. We found that the seconds that the model rats cost during the test was not significantly different between the control group and ZBPYR group (Fig. 3d). In addition, the number of times that the model group rats across the original platform was lower than that of control group (Fig. 3e, *p* < 0.05). However, there was no significant difference between ZBPYR group and model group. Performance in the visible platform version was similar in the three groups in terms of escape latency (Fig. 3f). All these findings suggest that ZBPYR could attenuate the impairment of learning and memory behaviors of the diabetic model rats.

### Effects of ZBPYR on mitochondrial ultrastructure changes

**Fig. 4:**
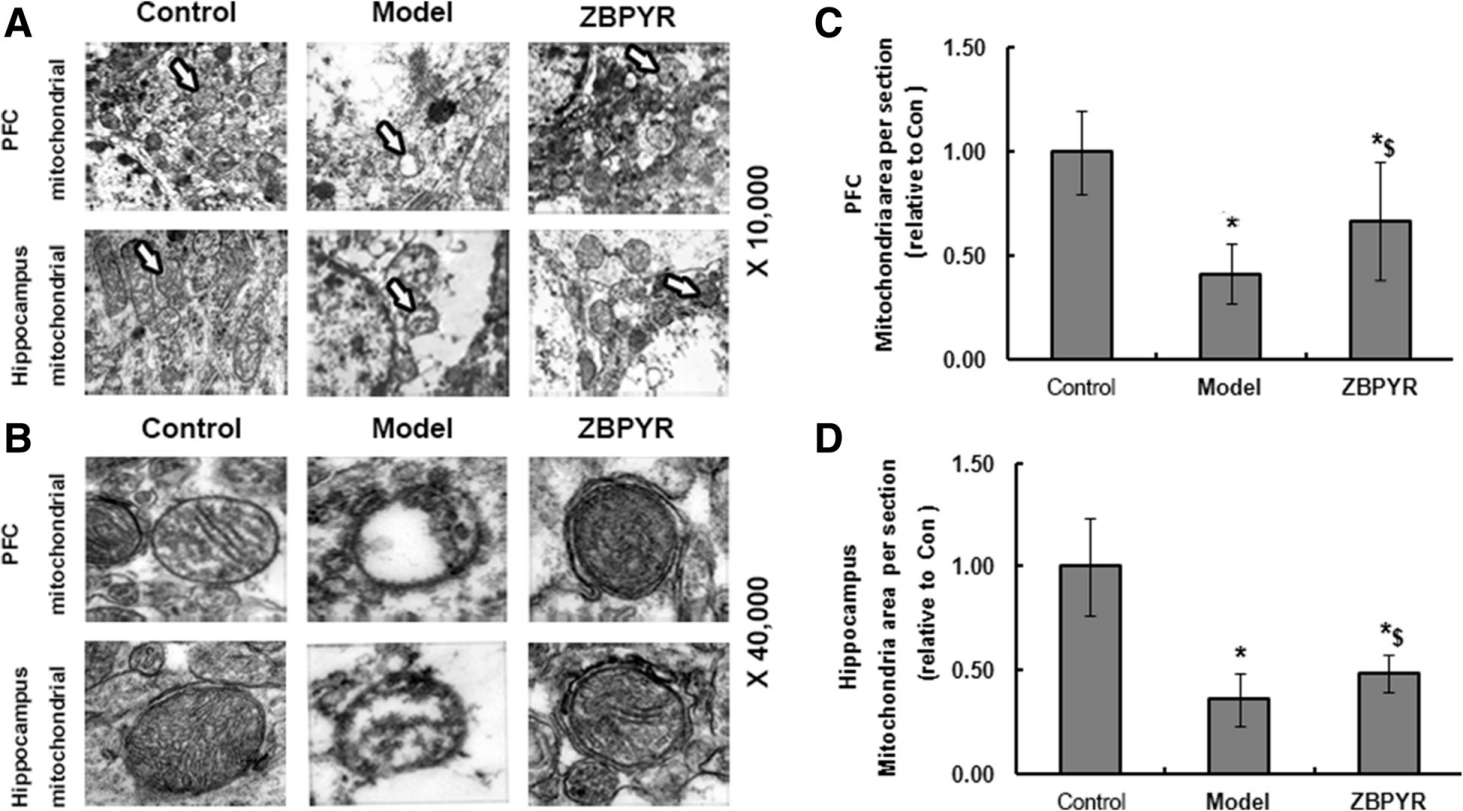
Alteration of mitochondria structure. TEM images at original magnifications of × 10,000 **a** and × 40,000 **b** in PFC and hippocampus mitochondria of rats in control, model and ZBPYR groups. **c** and **d** Quantification of mitochondria in per section (analysis of 5 images in 3 rats per group). Results were normalized by the mean value for control rats set to 1 unit. *Compared with Control group; ^$^ ZBPYR compared with Model group;*^, $^ *p* < 0.05, **^, $$^ *p* < 0.01

Marked alterations in mitochondrial morphology and the outcomes (×10,000) of PFC and hippocampus were shown in Fig. 4a. Higher magnification (×40,000) showed swelling of mitochondria associated with an increased number of disordered and partially disrupted cristae in the model group compared with the control group. However, less damage of mitochondrial ultrastructures in PFC and hippocampus was observed in the ZBPYR group compared with the model group (Fig. 4b). Additionally, in the model group, areas of both PFC and hippocampal mitochondria were lower (59 % and 64 %, respectively; *p* < 0.05) than that of the control group, whereas higher (26 % and 13 %, respectively; *p* < 0.05) in ZBPYR group than of model group (Fig. 4c and d).

### Effects of ZBPYR on mitochondrial transmembrane potential alterations

**Fig. 5:**
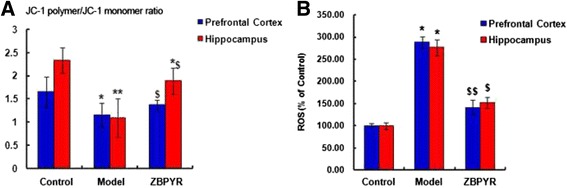
Effect of ZBPYR on ΔΨm and ROS production in the PFC and hippocampus tissues of rats in control, model and ZBPYR groups. **a** Ratios of JC-1 polymer to JC-1 monomer (red/green fluorescence). **b** The level of ROS production was monitored by the DCFH-DA assay. Data are expressed as the mean ± SD. *p < 0.05 and **p < 0.01 compared with Control group. *Compared with Control group; ^$^ ZBPYR compared with Model group;*^, $^ *p* < 0.05, **^, $$^ *p* < 0.01

The levels of ΔΨm were shown in Fig. 5a. In the control group, the ratio of JC-1 polymer/JC-1 monomer in PFC and hippocampus were 1.65 ± 0.33 and 2.33 ± 0.28, respectively. Compared with the control group, lower ratio in the model group (PFC: 1.15 ± 0.26, *p* < 0.05 and hippocampus: 1.09 ± 0.42, *p* < 0.01, respectively) indicated the dissipation of ΔΨm. ZBPYR group demonstrated the attenuated dissipation of ΔΨm (PFC: 1.36 ± 0.12 and hippocampus: 1.88 ± 0.28, respectively; *p* < 0.05). The differences between the ZBPYR group and the control group were only found in hippocampus tissues.

### Effects of ZBPYR on ROS Level

As shown in Fig. 5b, the increased ROS levels detected by DCFH assay were observed in the PFC and hippocampus of model groups (*p* < 0.05), and ZBPYR significantly inhibited the elevation of ROS levels with *p* < 0.01 in the PFC and *p* < 0.05 in the hippocampus.

### Effects of ZBPYR on neuron cytoskeleton proteins and biomarker of inner mitochondrial membrane proteins

**Fig. 6:**
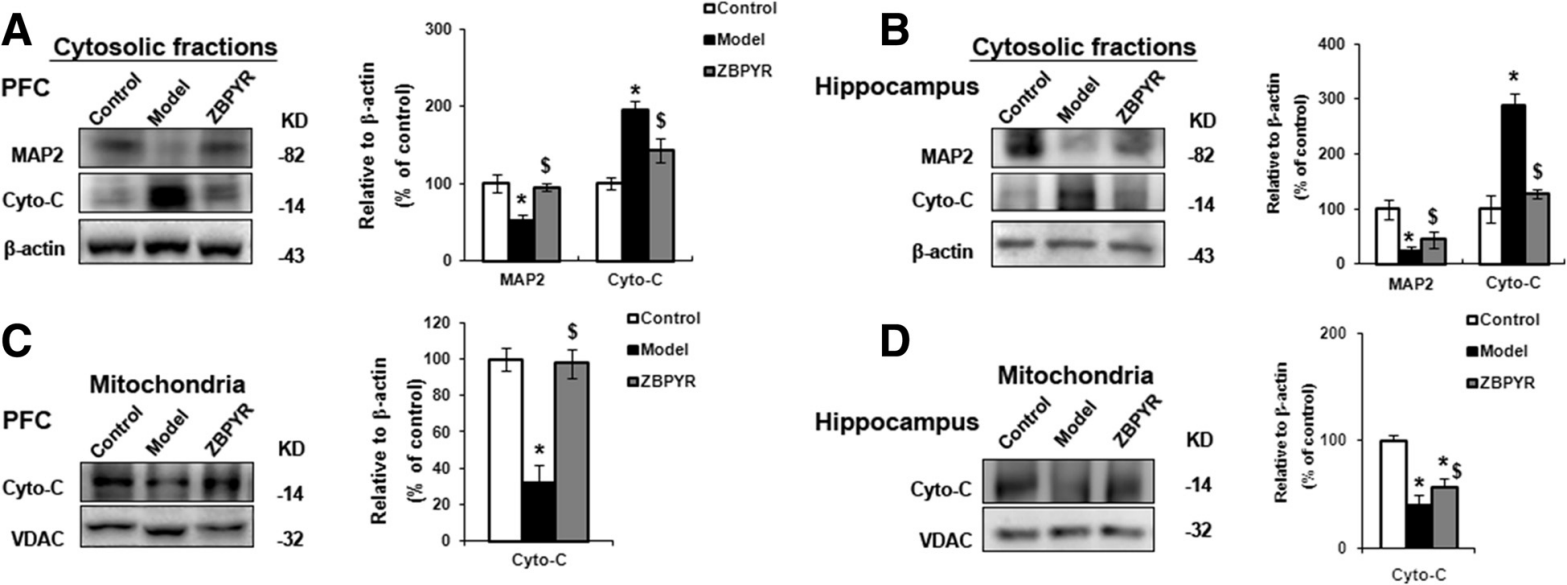
Effects of ZBPYR on cytochrome c (Cyto-C) and MAP2 levels. **a** and **b** ZBPYR significantly increased MAP2 expression and decreased Cyto-C expression in cytosolic fractions in PFC and hippocampus, respectively. **c** and **d** ZBPYR significantly reduced the impairment of Cyto-C in PFC and hippocampus, respectively. Representative Western Blot and bar graphs of gray-scale analysis were shown. β-action was used as a loading control of cytosolic fractions. VDAC was used as a loading control of mitochondria. Data were presented as mean ± SD. *p < 0.05 and **p < 0.01 compared with Control group. *Compared with Control group; ^$^ ZBPYR compared with Model group;*^, $^ *p* < 0.05, **^, $$^ *p* < 0.01

In the present work, the protein expression level of neuron cytoskeleton protein MAP2 was measured. As seen in Fig. 6a, MAP2 expression was obviously increased by ZBPYR compared with model group in PFC (*p* < 0.05). In addition, the activities of MAP2 were also significantly increased by ZBPYR in hippocampus (Fig. 5b, *p* < 0.05). Mitochondrial biomarker cytochrome c was also investigated in this work. Compared with model groups, the expression of cytochrome c in the cytosol were down-regulated by ZBPYR with *p* < 0.05 in PFC and hippocampus as shown in Fig. 6a and b, respectively. However, level of cytochrome c in the mitochondria was enhanced in the PFC and hippocampus by ZBPYR (Fig. 5c and d, *p* < 0.05), which indicated that the release of cytochrome c from mitochondria was improved after ZBPYR treatment.

### Effects of ZBPYR on insulin signaling and GSK3β

**Fig. 7:**
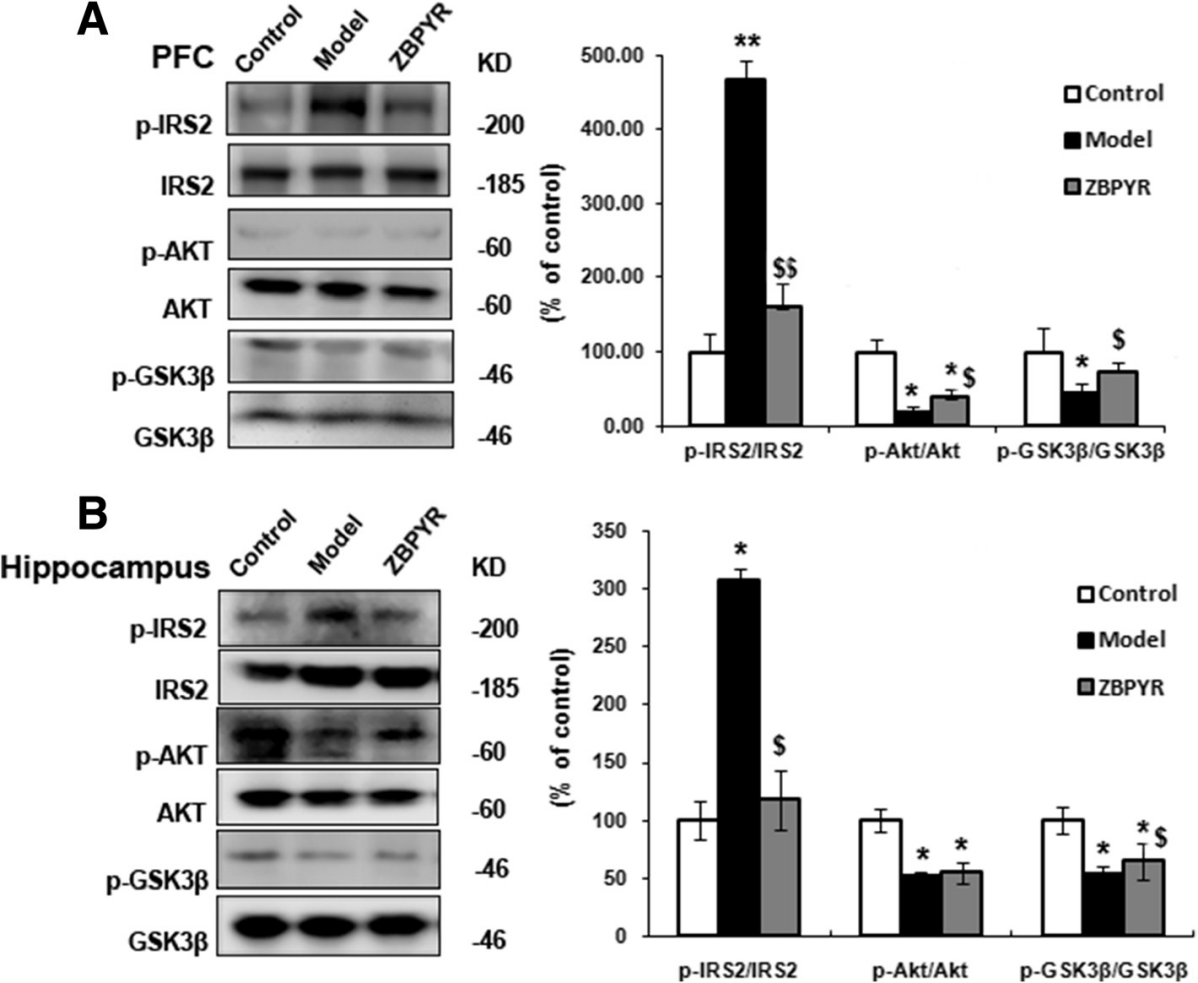
Effects of ZBPYR on insulin signaling and GSK3β activity. **a** and **b** ZBPYR reduced p-IRS2 at serine 731, increased p-Akt at serine 473 and significantly improved GSK3β activity by increasing p-Ser^9^ GSK3β expression in the PFC and hippocampus, respectively. Data are presented as mean ± SD. *Compared with Control group; ^$^ ZBPYR compared with Model group;*^, $^ *p* < 0.05, **^, $$^ *p* < 0.01

In order to evaluate the effects of ZBPYR on insulin signaling of DACD rats, the expressions of insulin signaling factor IRS2 and p-Ser^731^ IRS2, Akt and p-Ser^473^ Akt in PFC and hippocampus were evaluated by Western Blot analysis. As shown in Fig. 7a and b, no obviously changes of total levels of IRS2 or Akt before or after ZBPYR treatment were checked in any brain region among the three groups. However, we observed a significantly increased level of p-Ser^731^ IRS2 (*p* < 0.01) and apparently down-regulation level of p-Ser^473^ Akt (*p* < 0.05) in the PFC of model groups (Fig. 7a). In the same region of ZBPYR group, the expression of p-Ser^731^ IRS2 was markedly down-regulated (*p* < 0.01) and p-Ser^473^ Akt was elevated compared with model group (*p* < 0.05). Meanwhile, the proteins which were expressed in the hippocampal groups were also tested (Fig. 7b). These results indicated that compared with model groups, expression of p-Ser^731^ IRS2 was significantly down-regulated with *p* < 0.05 and p-Ser^473^ Akt was not changed apparently by ZBPYR (*p* < 0.01).

GSK3β was a well known down-stream target of insulin signaling [20, 21] and we tested the expression of GSK3β in our model. It was shown that there was no change in the total level of GSK3β in PFC and hippocampus (Fig. 7a and b). However, a down-regulation of p-GSK3β expression level at p-Ser^9^ was found in the PFC and hippocampus of model groups (*p* < 0.05) and ZBPYR increased the p-GSK3β level compared with the model groups (*p* < 0.05).

### Effects of ZBPYR on the expressions of Aβ_1-42_ and MAP2

**Fig. 8:**
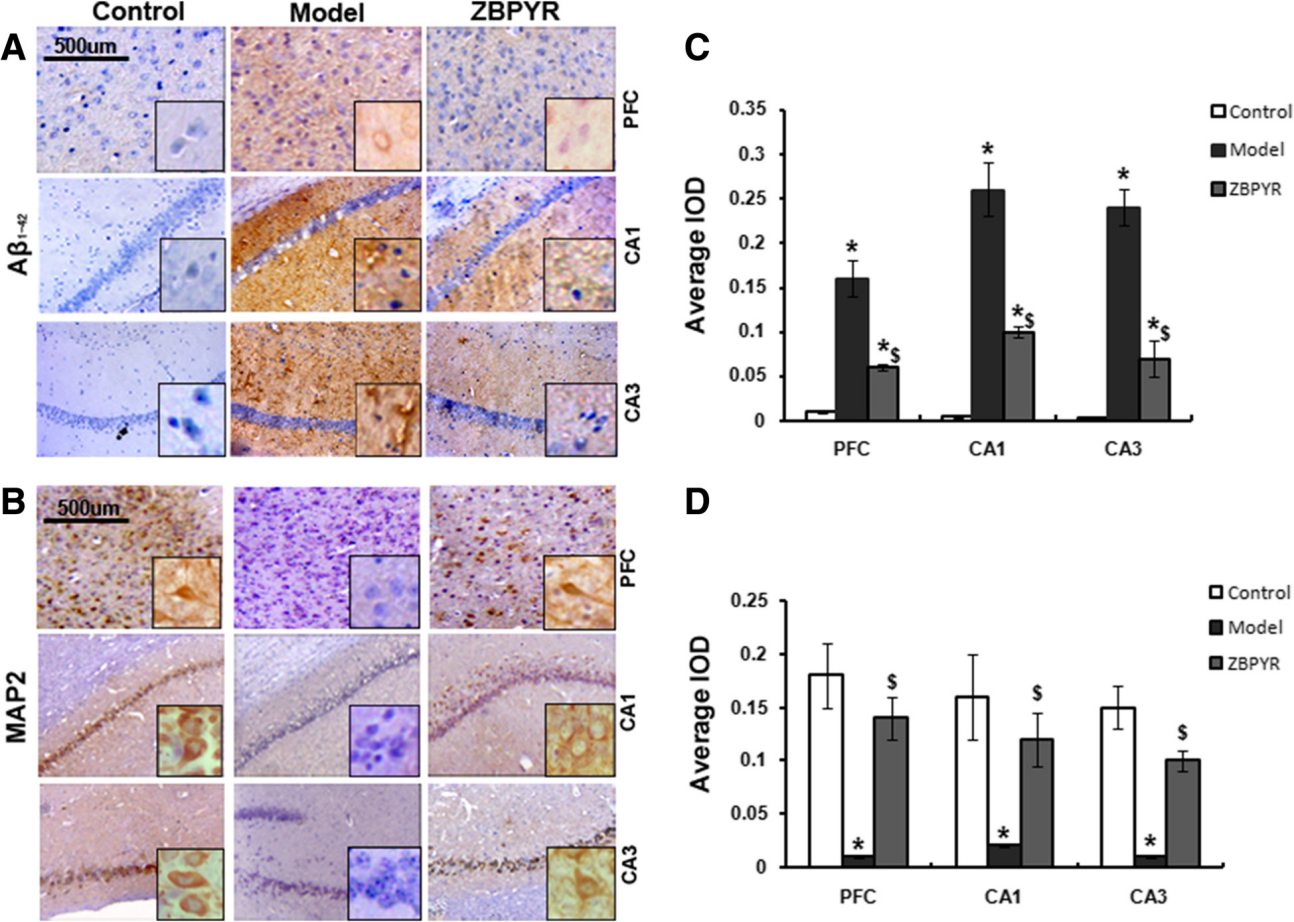
Effects of ZBPYR on Aβ_1-42_ and MAP2. **a** Immunohistochemical staining (the main images, x50; inset boxes, x200) of Aβ_1-42_ in the PFC, hippocampus CA1 and CA3. **b** Immunohistochemistry staining (the main images, x50; inset boxes, x200) of MAP2 in the PFC, hippocampus CA1 and CA3. **c** and **d** The average IOD of the immunoreactive area for Aβ_1-42_ and MAP2 was presented as bar graphs. Each bar represents mean ± SD of three independent experiments (*Compared with Control group; ^$^ ZBPYR compared with Model group;*^, $^ *p* < 0.05, **^, $$^ *p* < 0.01)

In this work, the expression of Aβ_1-42,_ which was the characteristic of DACD, was detected [22]. The results indicated that Aβ_1-42_ was observed almost negative in the PFC, hippocampus CA1 and CA3 of control groups. Additionally, the increased expression of Aβ_1-42_ was obviously detected in model groups in the different brain areas (PFC: 16 times, CA1: 52 times and CA3: 60 times). In addition, Aβ_1-42_ expression was significantly decreased by ZBPYR with PFC: 0.37 times, CA1: 0.38 times and CA3: 0.29 times compared with model groups (Fig. 8a and c). There was no significant change in the hippocampal DG (dentate gyrus) among the three groups (data not shown). Furthermore, the biomarker of dendritic cytoskeleton MAP2 was also investigated using immunohistochemistry staining (Fig. 8b). The expression of MAP2 was significantly decreased in the PFC (0.05 times), hippocampus CA1 (0.1 times) and CA3 (0.06 times) of model groups when compared with control groups. And we found the expression of MAP2 was significantly increased after ZBPYR treatment in these three areas (PFC: 14 timesl, CA1:6 times, CA3: 10 times).

## Discussion

DACD is considered as a common neurodegenerative disease, which lacks an efficient therapeutic regimen [23–25]. The high efficiency and low toxicity of TCM, which has the advantage of providing multiple therapeutic effects on multiple targets, suggest the feasibility in the treatment of DACD. TCM ZBPYR plays crucial roles in learning and memory impairment in the diabetic rats through inhibiting endoplasmic reticulum stress, maintaining the morphology of dendritic spines and ameliorating DACD in proteomic analysis [7, 14, 16]. The individual herbs used in ZBPYR exhibited some pharmacological effects. For example, ginsenosides in *Panax ginseng* C.A.Mey reduced cerebral ischemia-induced tau phosphorylation and attenuated the symptoms of DACD [26, 27]. Additionally, senegenin from *Polygala tenuifolia* Willd processed neuroprotective potential against Aβ-induced neurotoxicity [28]. It was also found that β-asarone in *Acorus gramineus* Sol. ex Aiton had the therapeutic potential against high-fat diet induced obesity in rats [29]. These compounds may contribute to the effects of ZBPYR in preventing diabetes or neuroprotection. In spite of that, the mechanism of ZBPYR in regulating energy metabolism is still unknown. Mitochondria play a central role in energy metabolism. In the brain neurons, glucose is metabolized by mitochondria to produce cellular energy. It has been reported that mitochondrial dysfunction existed in multiple peripheral tissues of diabetic rats [30–32]. However, the mitochondrial functions in DACD animals were still unclear. The aim of this study was to investigate the function of brain mitochondria in high fat diet combined with STZ induced DACD rats and its related mechanisms of the protective effects of ZBPYR.

In our study, high fat diet combined with STZ injection rat model showed hyperinsulinaemia and hyperglycaemia, indicating that the diabetic model was successfully built. In addition, the rat model registered cognitive decline by MWM. ZBPYR treatment could restore the learning and memory retrieval behaviors induced by diabetes.

The energy of brain mainly depends on mitochondria. Maintaining mitochondrial function is essential for neuron survival and offers protection against neurodegeneration [33–36]. Here, we investigated the amount, structure, and function of mitochondria in the DACD rat brain. Our data indicated that the changes in mitochondrial density and structure by TEM were observed in the region of PFC and hippocampus of DACD rats. In addition, loss of ΔΨm and increased production of ROS were also found in the model groups, which suggested that mitochondria dysfunction existed. ZBPYR could alleviate the changes in the structure and increase the number of the mitochondria in per area. It was also found that mitochondrial membrane potential was pronounced and the level of ROS decreased in the same region of brain after ZBPYR treatment. Taken together, these results proved that mitochondria dysfunction was the mean target of ZBPYR. Accumulative evidence suggests that alterations in mitochondrial structure and function are associated with insulin signaling and related complications [37–41]. Moreover, recent epidemiological evidence suggests that CNS insulin resistance is a risk factor for cognitive decline [42–44]. Downstream targets of insulin signaling pathway, e.g., insulin receptor substrate serine phosphorylation 2 was up-regulated in the brains of obese rats [45]. Akt, a key marker protein of insulin signaling, mediates the effect of insulin via important intracellular signaling cascades including the PI3K/Akt pathway [46, 47]. This work identified increase in p-IRS2 and decrease in p-Akt in the PFC and hippocampus of model rats, suggesting that central insulin signaling was impaired. ZBPYR could correct CNS insulin resistance by regulating p-IRS2 and p-Akt. In addition, ZBPYR exhibited strong activity in the inhibition of GSK3β. GSK3β is a downstream substrate of Akt and is the bridge connecting insulin signaling and Aβ, since the phosphorylation states of GSK3β in the brain were found to be involved in fluctuations of Aβ deposition [48–50]. In this study, we found that GSK3β activity was inhibited after ZBPYR treatment. Taken together, the present findings provide molecular biological evidence for the preventive effects of ZBPYR on DACD.

Amyloid β was reported to be the predictor of dementia and Aβ_1-42_ accumulation was found more obvious in the brain of cognitive decline patients [51–53]. Our previous study indicated that Aβ_1-42_ was down-regulated in db/db mice after ZBPYR treatment [16]. In the present results, Aβ_1-42_ accumulation was more apparent in model group in the region of PFC and hippocampus (CA1 and CA3) and less detectable in ZBPYR group. Then, the density of dendritic marker MAP2, which reflected the changes of the neuronal morphology, was significantly decreased in either brain region of the models. ZBPYR was efficacious for alleviating the decreases in MAP2 levels, particularly in the PFC and hippocampus (CA1 and CA3). We proposed that Aβ_1-42_ accumulation and low expression of MAP2 in brain were the reason for DACD. ZBPYR significantly enhanced the learning ability of the DACD rats, which is largely due to the decrease in Aβ_1-42_ level and increase in MAP2 expression.

## Conclusions

The findings of this study suggested that ZBPYR could prevent the brain impairment of DACD rats. The related mechanisms might be associated with improving mitochondrial dysfunction, insulin resistance and pathological changes including expressions of Aβ and MAP2.

## Abbreviations

Akt, protein kinase B; BCA, bicinchoninic acid; CNS, central nervous system; DACD, diabetes associated cognitive decline; FSI, fasting serum insulin; IOD, integrated optical density; IRS, Insulin receptor substrate; ITT, insulin tolerance test; MAP2, microtubule-associated protein 2; MWM, morris water maze; OGTT, oral glucose tolerance test; PB, sucrose solution; PFC, prefrontal cortex; RBG, red blood glucose; ROS, reactive oxygen species; STZ, Streptozocin; T2DM, type 2 diabetes mellitus; TCM, traditional Chinese medicine; TEM, transmission electron microscopy; VDAC, Voltage-dependent anion channel; ZBPYR, ZiBu PiYin recipe; ΔΨm, mitochondrial membrane potential
